# Comparison of Neck Circumference, Waist Circumference, and Skinfold Thickness in Measuring the Subcutaneous Fat Distribution and Their Association with Handgrip Strength: Cross-Sectional Study

**DOI:** 10.3390/ijerph192114283

**Published:** 2022-11-01

**Authors:** Faisal Asiri, Snehil Dixit, Saud F. Alsubaie, Kumar Gular, Adel Alshahrani, Ravi Shankar Reddy, Ajay Prashad Gautam, Jaya Shanker Tedla

**Affiliations:** 1 Department of Medical Rehabilitation Sciences, College of Applied Medical Sciences, King Khalid University, Abha 62529, Saudi Arabia; 2 Department of Physical Therapy and Health Rehabilitation, College of Applied Medical Sciences in Al-Kharj, Prince Sattam bin Abdul-Aziz University, Al-Kharj 11942, Saudi Arabia; 3 Physical Therapy Program, Health Rehabilitation Department, College of Applied Medical Sciences, Najran University, Najran 11001, Saudi Arabia

**Keywords:** cardiovascular disease, handgrip strength, neck circumference, handgrip asymmetry, waist to height ratio

## Abstract

Skinfold measurement (SKF) can accurately measure abdominal obesity and is regarded as a surrogate marker to predict non-communicable diseases. The objective of the present study was to observe the degree of association between neck circumference (NC), SKF and handgrip strength (HGS). Secondly, also to know the effects of smoking on NC, HGS and SKF. The mean and standard deviations and frequencies in percentage were analyzed, respectively. The degree of association between NC, anthropometric characters and HGS was also analyzed using the Pearson correlation. Furthermore, multiple linear regression models were used to study the degree of influence of independent variables on dependent variables. Correlation assessment for neck circumference with waist circumference and HGS revealed a weak association. While with SKF for four sites, a strong association was found. A significant regression was found among the smokers in the model (F (2, 7) = 5.2, *p*-value of 0.04 with an R2 of 0.598). The predictor variables, like waist and NC, can produce a variation of 59.8% in the dependent variable. Whereas, among non-smokers, an insignificant regression was seen. In conclusion, neck circumference is associated with SKF. However, a small sample size of young smokers revealed that NC and waist circumference influenced HGS.

## 1. Introduction

The global burden of non-communicable diseases is constantly increasing worldwide [1]. The contribution of obesity to the worldwide disease burden of cardiovascular disease, hypertension, and mortality is on the rise [1]. In Europe alone, non-communicable disease accounts for an increased percentage of the disease burden, amounting to 71%, with a death rate of 86% [2].

Research focusing on adipose tissue has taken a front stage in recent years [3]. Usually, the fatty tissue covering the internal organs is termed visceral adipose tissue (VAT), while the fatty tissue just below the surface of the skin is termed subcutaneous adipose tissue (SAT) [3]. It is now clinically well-established that many health risks are associated with abnormal VAT and SAT accumulation, including metabolic syndrome, hepatic steatosis, insulin resistance, and hypertension. However, the proliferation of adipose tissue might be regulated by biological, genetic and lifestyle aspects [3].

Upper body fat accumulation may carry an increased hazard for cardiometabolic risk factors and subcutaneous atherosclerosis [4]. As evaluated by neck circumference, the fat in the upper part of the body may indicate hazard levels more than visceral abdominal fat accumulation [4]. There is a direct link between free fatty acid levels and upper-body fat, signifying a significant association between the pathogenesis risk factor [4,5]. Neck obesity can predict moderate to high chances of developing cardiovascular risks than other anthropometric measures [6]. Neck circumference (NC) has also been identified as a novel indicator for hyperuricemia. A categorization value limit for neck circumference was 39.1 cm in males and 35.1 cm in females in a study which was consistently associated with hyperuricemia even with standard limits of glycated hemoglobin and blood pressure in the Asian Chinese population [7].

Additionally, simple and conventional methods of anthropometric evaluation, like skinfold thickness (SKF) evaluation, are believed to have a predictive value for central obesity and non-communicable diseases like diabetes [8]. Truncal skinfold thickness is more strongly linked to insulin resistance than abdominal visceral adiposity as measured by magnetic resonance imaging (MRI) [8]. Moreover, because of its ease of use, cost-effectiveness and non-invasive qualities, it is commonly recommended in clinical and epidemiological studies [9].

Moreover, handgrip strength (HGS) has received considerable focus and attention in research. HGS can indicate muscle strength, muscle mass, physical fitness and overall health [10]. HGS can be influenced by a variety of factors like cardiometabolic risk factors and smoking status [11]. Research has stated that individuals with smoking habits have an inverse relationship between HGS and cardiometabolic risk factors [11]. Another study also found an association between HGS among smokers with higher hospitalization rates among Japanese males with type 2 diabetes [12].

A decrease in HGS can be directly correlated with malnutrition, falls, lengthier hospital stays, decreased quality of life, and higher chances of frailty and death rate in health and population with disease [10]. Hence, HGS has been extensively used as a recommended way to clinically stratify individuals with a high risk of mortality and morbidity [10]. A study exploring the effect of low HGS in adolescents concluded that after 24 years, there are higher rates of death in adulthood due to suicide and cardiovascular disease [13]. Additional co-factors like the presence of smoking may be further associated negatively with muscle strength [14].

Hence, there is a need to know whether neck circumference is associated with health indexes like HGS and to understand its relationship with SKF. Therefore, the objective of the present study was to see the degree of association between NC, SKF and HGS. Secondly, also to know the effects of smoking on NC, HGS and SKF.

## 2. Materials and Methods

The study is registered with clinicalTrials.gov (accessed on 7 September 2022) with the identifier number NCT05231291, with ethical approval number ECM#2021-3603. Before the commencement of the study, informed consent was obtained from the participants. The participants were then randomly nominated using probability sampling. In this cross-sectional study, 39 male participants were registered from the college, and an assessment was performed. The included participants were from a healthy population with no previous history of chronic diseases (*n* = 39).

The sample size in the study was determined by the formula based on ISO general standards for the age group of 18–29 years only from a previously published study in the same population [15]. Hence, the sample for the current study came to 74.

**Figure 1:**
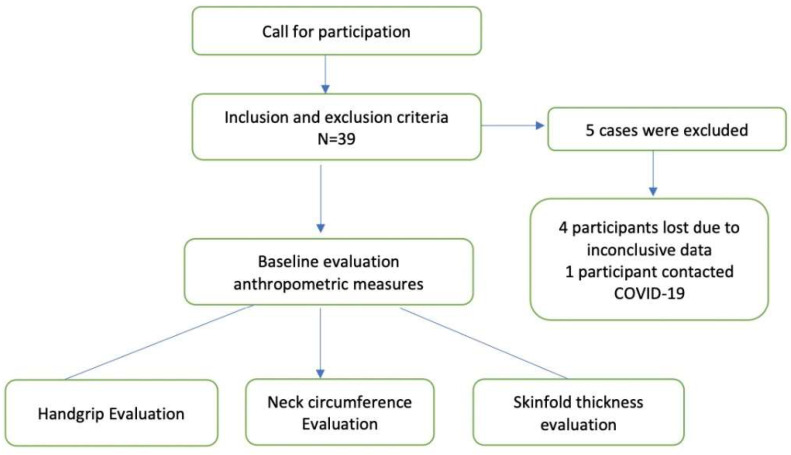
Flow of participants in the study.

The exclusion criteria were if students did not give their consent to participate, previous history of thyroid diseases (*n* = 0), chronic disease state (*n* = 0), acute musculoskeletal injury. Subjects taking medicine for obesity, dyslipidemia (*n* = 0) or weight control in any form which may affect the test, the participant’s having a difficult time to follow the tests’ instructions even after familiarity session (*n* = 0), lack of data (*n* = 4) were also excluded. In addition, students reporting any acute infections and a previous history of acute critical respiratory illness were also excluded (*n* = 1). Finally, only *n* = 34 participants were considered for the study. Figure 1 depicts the flow of participants in the study.

*Adiposity Measurements*

*Neck circumference and anthropometric measurement:* Neck circumference (NC) was measured in centimeters (cm) using a tape (*n* = 34). Participants stood in an erect position with their heads in the Frankfort horizontal plane. The upper margin of the measuring tape was placed under the cricothyroid cartilage perpendicular to the neck axis [16]. Waist circumference was measured according to the criteria laid by the world health organization (WHO). Height was measured using a stadiometer.

*Waist-to height-ratio (WHtR):* was calculated using waist in centimeters (cm) divided by height in cm. A value of 0.5 was used to identify people with a premature risk linked with central obesity using a value of 0.5 and above. WHtR was classified as: ‘no increased risk’ (WHtR < 0.5), ‘increased risk’ (WHtR ≥ 0.5 and < 0.6) and ‘very high risk’ (WHtR ≥ 0.6) [17].

*Handgrip strength measurement (HGS):*

Hydraulic Dynamometers by Jamar were used to evaluate the handgrip strength (Hydraulic Hand Dynamometer Fabrication Enterprises Inc, New York, NY, USA). The examiners confirmed that the Jamar handgrip dynamometer was well calibrated before the test. The participants’ HGS examination protocol was conducted as per the recommendations stated by the National Institute of Health Research (NIHR) [18].

Beforehand the participants were shown how to hold the dynamometer. The norms were for participants to sit with a good trunk and hand support posture. The forearm was held mid-prone, wrist free from the chair’s armrest, and thumb pointing up. In addition, before every measurement, it was ensured that the needle was at zero [16].

The HGS evaluation was first done on the right hand, trailed by the left. Recommended sentences were utilized for encouragement to squeeze the handle for the best possible results [16].

*HGS Asymmetry*: the highest recorded HGS values from the non-dominant and dominant hand were considered to calculate the HGS ratio (non-dominant HGS (kg)/dominant HGS (kg)). The “10% rule” states that HGS asymmetry is said to be present when the participants have an HGS ratio less than 0.90 (i.e., 10%), indicating asymmetric dominant HGS, whereas those with an HGS ratio > 1.10 (i.e., 10%) were categorized to have asymmetric non-dominant HGS [19]. Those with an HGS ratio < 0.90 or > 1.10 were considered to have asymmetric HGS, while those with an HGS ratio 0.90–1.10 had HGS symmetry [20].

*Skinfold thickness (SKF) measurement:* The locations used for measuring skinfold thickness (SFT) were biceps, triceps, and subscapular and suprailiac on the non-dominant side. An expert investigator evaluated it by marking at multiple sites using a Harpenden Caliper (Baty, UK). SKF was assessed at different sites each time by carefully observing the places. It was made sure that the instrument was well calibrated before performing the test. The third measurement was mandatory if the minimum difference between the estimated sites exceeded 3 mm [17]. The body fat percentage was obtained by using the mean values of the two nearest assessments as in the formula stated by Durnin and Womersley [17,18]. Siri’s formula ((4.95/B.D.) − 4.50) × 100) was used to obtain the body fat percentage [19]. The fat mass (FM, kg) and fat-free mass (FFM, kg) was computed as (%F × weight)/100 and weight—FM, respectively.

## 3. Statistical Analysis

The mean and standard deviation were presented as descriptive statistics in Table 1. Frequency data were also used for categorical data. All the parameters were reported in the International System of Units (SIs). As the data was skewed, natural log transformation was used to transform skewed data to nearly conform to the normality of distribution. Values were later back-transformed using antilog.

One-way ANOVA was applied to recognise any interaction effects between the independent and the dependent variables. The significance of the study was fixed at *p* < 0.05. The statistical test, one-way ANOVA, was conducted for the outcomes by employing a statistical package for the social sciences (SPSS 26, IBM, Armonk, NY, USA). Furthermore, the results were reported as degrees of freedom (Df1, Df2), *F*, and *p* values.

The relationship between anthropometric variables and grip strength was explored using the Pearson correlation coefficient. Pearson correlation for Neck circumference, waist, handgrip strength, and skinfold measures (biceps, triceps, subscapularis, and suprailiac) was done.

Further, the multiple linear regression analysis explored the associations between dependent and independent variables. Finally, multiple regression analysis using the enter method was carried out to assess possible predictors. The degrees of freedom (Df), *F* value, *p*-value, and R^2^ values were reported for the multiple linear regression analysis. For the study.

## 4. Results

**Figure 2:**
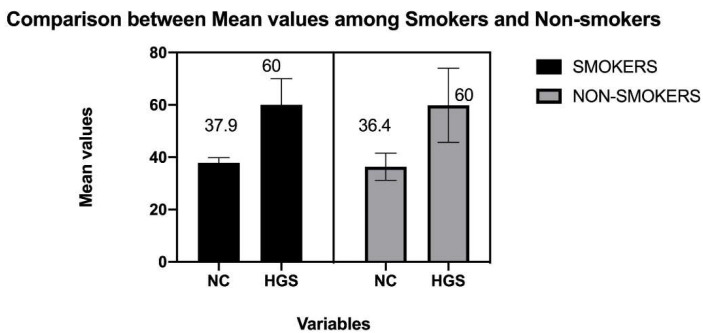
Comparison between mean values among smoker and non-smoker for neck circumference (NC) and handgrip strength (HGS).

**Figure 3:**
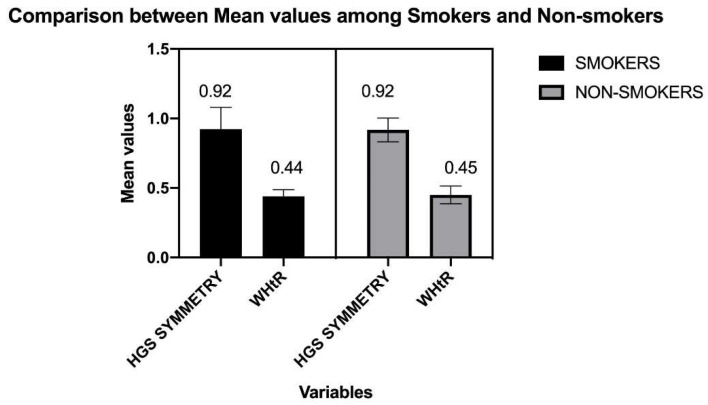
Comparison between mean values among smokers and nonsmokers for HGS symmetry waist to height ratio (WHtR).

The standard descriptions of the participants are mentioned in Table 1 and Table 2. Figure 1 shows the flow of participants in the study. The mean values of HGS, NC, WHtR, and HGS symmetry are compared in Figure 2 and Figure 3, respectively.

*Correlation assessment* for neck circumference with waist circumference revealed a *r*-value of 0.298 and *p* = 0.09, for HGS *r* = 0.357 and *p* = 0.04, for SKF measurement biceps *r* = 0.220 and *p* = 0.308, triceps *r* = 0.180 and *p* = 0.308, Subscapularis *r* = 0.312 and *p* = 0.073 and Suprailiac *r* = 0.385 and *p* = 0.025. The correlation between NC and waist circumference was not significant, but with HGS, it was significant. Correlation of waist with SKF biceps had a *r* = 0.785 and *p* = 0.00, with triceps *r* = 0.810 and *p* = 0.00, with subscapularis *r* = 0.813 and *p* = 0.00, for Suprailiac *r* = 0.728 and *p* = 0.00 was found.

*Regression analysis:* Multiple regression equations indicated the association between the dependent variable of HGS and independent variables like waist and neck circumference among non-smokers and smokers. Enter method was used for the analysis. A significant regression was found among the smokers in the model (*F* (2, 7) = 5.2, *p*-value of 0.04 with an R^2^ of 0.598). The predictors’ variables, like waist and neck circumference, can produce a variation of 59.8% in the dependent variable. Whereas among non-smokers for the same model, a non-significant regression was found (*F* (2, 20) = 0.17, *p*-value of 0.85 with an R^2^ of 0.017).

*Interaction analysis:* There statistical difference was insignificant between the dependent (waist circumference, neck circumference, HGS) and (smoking) independent variables was determined by the between-group differences using one-way ANOVA, for neck circumference (*F* (1,32) = 0.52 *p* = 0.478), for waist (*F* (1,32) = 0.002 *p* = 0.969), for HGS (*F* (1,32) = 0.097 *p* = 0.757). Hence, no interaction effect of smoking on the dependent variables was observed.

## 5. Discussion

Neck circumference is the new paradigm for evaluating cardiometabolic disease [21]. Even in patients with low cardiometabolic risk, a larger neck circumference was linked with a larger epicardial fat thickness [22]. In a study, the agreed categorisation for neck circumference was found to be 39.25 cm in the Asian population for evaluating subjects with central obesity with a sensitivity of 89% and specificity of 71% [23]. Furthermore, these values for neck circumference have shown a significant increase in risk for non-communicable diseases [23].

In the current study, neck circumference revealed a low correlation between waist circumference and HGS. At the same time, a high positive correlation was found for SKF for four sites (biceps, triceps, subscapularis and Suprailiac). The results from the Framingham heart study indicate that subcutaneous fat distribution in the upper parts of the body may be suggestive of pathology [24]. In addition, previous studies have found a strong correlation between NC and visceral adipose tissue [24]. Hence, NC indicates the subcutaneous fat distribution and may mean visceral fat deposits.

Handgrip strength is an easy, speedy, and economical method to measure muscle strength. Apart from muscular strength, it’s also an indicator of weakness and a robust forecaster of all-cause mortality [25]. Previous follow-up studies have testified that handgrip strength is strongly linked with myocardial infarction, cardiovascular deaths and stroke [25]. In the present study, HGS was found to be influenced significantly by waist and neck circumference among smokers 60%, whereas such influence among non-smokers was not observed. In addition, reduced handgrip strength was reported in previous studies among smokers compared to non-smokers with quick fatigability compared to non-smokers [26]. Although no interaction, effect was seen when using smoking as an independent factor.

An asymmetric HGS ratio is between <0.90 or >1.10, while those with an HGS ratio of 0.90–1.10 have HGS symmetry [20]. In the present study, right-side dominance participants had 0.92 ± 0.11, and left-side dominance had values of 1.01 ± 0.09. However, the participants in the present study had values within the HGS symmetry. However, the values of HGS symmetry appear to be more on the borderline risk for the provided cut-off. On the contrary, handgrip asymmetry is often associated with a future risk of cardiac and neurodegenerative disorders [27,28]. Furthermore, a study analyzing the participants from 2006–2014 found that HGS asymmetry may independently be connected with the risk of accelerated death rates [28].

Another anthropometric measure, WHtR, has been associated with the risk of hypertension among the Korean population [26]. WHtR is better predictive of cardiac events than other anthropometric measures. The agreed cut-off around the globe for the adult population has been around 0.5–0.56 in men [17,28]. In the present study, even though it comprised healthy participants, the WHtR values (0.45) were quite close to the cut-off of 0.5, implying an ‘iceberg phenomenon’ in general medical practice, meaning most cases may remain subclinical [28].

## 6. Strength and Limitation

The relationship between HGS, NC and SKF was explored. HGS symmetry and WHtR were also studied, where values were found to be a borderline risk, which may imply a larger subclinical problem. However, the study had limitations as the study might have suffered a few methodological biases due to the COVID-19 situation. In addition, the study saw a lower participation level among the younger Arabic population, and a postulated reason could be lesser awareness among the people regarding the health utility of HGS as compared to the European counterparts.

## 7. Conclusions

Neck circumference is associated with SKF; however, it showed a weak association with waist circumference and HGS. Among smokers, the neck and waist circumference influenced HGS. However, no significant interaction was seen between smokers and non-smokers with the anthropometric measures. It was also observed that HGS symmetry and WHtR values were borderline among the young healthy participants. This might be indicative of a subclinical phenomenon though our understanding for the same is limited by the smaller sample size.

## Figures and Tables

**Table 1:** Characteristics of the study participants.

Variable (*n* = 34)	Mean ± SD
Age	21.44 ± 1.89
Smoking	Yes = 10; No = 24
Dominance	Right = 32; Left = 2
Height (cm)	170.59 ± 5.93
Neck circumference (cm)	36.82 ± 4.5
Waist circumference (cm)	76.24 ± 10.05
Average handgrip strength (Right)	64.1 ± 13.57
Average handgrip strength (Left)	58.78 ± 13.73
Total handgrip strength	58.5 ± 12.12
WHtR (Waist to height ratio)	0.45 ± 0.06
HGS symmetry (Right Dominance)	0.92 ± 0.11
HGS symmetry (Left Dominance)	1.01 ± 0.09

**Table 2:** Mean value for skinfold thickness assessment for four sites.

Variable (*n* = 34)	Mean ± SD
Biceps	3.5 ± 1.62
Triceps	5.57 ± 1.93
Subscapularis	5.78 ± 2.06
Suprailiac	4.96 ± 1.99
Body density	1.07 ± 0.01
Body Fat percentage	12.24 ± 5.16

## Data Availability

The data of the present study is available on adequate request as per the research policies of the King Khalid University.
